# 

**Published:** 1899-08-19

**Authors:** 


### NOTICE TO CORRESPONDENTS.

All MS., Letters, Books for Review, and other matters intended for the Editor should be addressed THE EDITOR, "THE HOSPITAL," THE HOSPITAL BUILDING, 28 & 29, SOUTHAMPTON STREET, STRAND, W.C.
The Editor cannot undertake to return rejected MS., even when accompanied by stamped directed envelope.

**Notice to Advertisers and Subscribers.**

All Advertisements, Orders for Copies of THE HOSPITAL, and Business Communications should be addressed to **THE MANAGER (Not to the Editor)**, THE HOSPITAL, The Hospital Building, 28 & 29, Southampton Street, Strand, London, W.C.
The Cost of Subscription, post free, is as follows:—Per week, 2½d.; per quarter, 2s. 8d.; per half-year, 5s. 3d.; per annum, 10s. 6d. Foreign:—Per annum, 15s. 2d., payable, in all cases, in advance.

**NOTICE.—Vol. XXV. of "The Hospital" (October, 1898, to March, 1899), in handsome cloth binding, is now on sale at the Office of this Journal, price 7s. 6d. Cases for binding the Numbers contained in this Volume 1s. 6d. Index to Vol. XXV., 2½d., post free.**
**The Monthly Part for September will be ready on September 1. Price 6d., by post 8d.**

### The Student of Medicine.

### APPOINTMENTS.

N. G. Bennett, B.A., B.C.Cantab., L.D.S., appointed Dental Surgeon to the Great Northern Central Hospital. C. M. Fegen, L.R.C.P.Edin., M.R.C.S.Eng., appointed Medical Officer of Health for the Croydon Rural District. H. A. Gunther, M.B., appointed House Surgeon to the Great Northern Central Hospital. T. J. Horder, M.B., B.Sc.Lond., appointed Physician to Out-patients at the Great Northern Central Hospital. F. Lonnon, M.R.C.S., L.R.C.P., L.D.S.Eng., appointed Dental Surgeon to the Gordon Boys' Home. W P. Purvis, B.Sc., M.D., M.S.Lond., F.R.C.S.Eng., appointed Surgeon in Charge of the Ear and Throat Department of the Royal South Hants Infirmary, Southampton. A. W. F. Sayres. M.D Brux., M.R.C.S.Eng., L.R.C.P. Lond., appointed Public Vaccinator for the Woodford District of the West Ham Union. W. M. Stevens, M.D.Lond., appointed Honorary Pathologist to the Cardiff Infirmary. H. Stratford, M.B., M.R.C.S.Eng., L.R.C.P.Lond., appointed Assistant House Surgeon to the Hereford General Infirmary. R. T. Williamson, M.D.Lond., M.R.C.P., appointed Honorary Physician to the Ancoats Hospital, Manchester. C. A. Young, M.R.C.S.Eng., L.R.C.P.Lond., appointed Medical Officer and Public Vaccinator for the No. 3 District of the Bridlington Union.

### THE ROYAL ARMY MEDICAL CORPS AND HER MAJESTY'S INDIAN MEDICAL SERVICE.

Papers Set at the Examination of Candidates—(*continued*).

### SURGERY.—July 28th, 1899.

(All four questions to be answered.)

1. How is a dislocation backwards of the hip joint produced? Give the diagnosis and treatment of the injury.
2. What are the different forms of cystic disease met with in the female mamma? Give the pathology, symptoms, and treatment of each variety.
3. For what conditions may iridectomy be required? Describe the operation, and give the after treatment of a case.
4. State fully the considerations which would influence your decision as to the treatment, either by amputation or by excision, of a case of tuberculous disease of the knee joint in a young adult.

### ANATOMY AND PHYSIOLOGY.—July 29th, 1899.

1. Describe the fascia of the psoas muscle, the fascia iliaca, and the fascia transversalis, laying particular stress upon those connections which bear upon the anatomy of psoas abscess, and of femoral and inguinal hernia.
2. Describe "Hunter's Canal," and state clearly the relative position of the parts contained within it.
3. Give the form, position, and relations of each suprarenal body, and mention what you know of its function.
4. Describe the optic nerve, the optic chiasma, and the optic tract, and state the central connections of the fibres which form the optic tract.

### CHEMISTRY AND MATERIA MEDICA.—July 29th, 1899.

1. What is the composition of chloroform? How is it prepared?
2. State the composition, and explain, with formulæ, the chemical preparation of (1) sulphuric acid, (2) hydrochloric acid, (3) nitric acid, (4) carbolic acid.
3. What rules regulate the strength of tinctures in the latest edition of the British Pharmacopœia? Give examples.
4. What is opium? What alkaloids does it contain? What are its official preparations, and what the strength of each?
5. What are the therapeutic uses of mercury and of its salts? What are their official preparations and doses?

### NATURAL SCIENCES.—August 4th, 1899.

(Candidates may answer not more than six questions, and they must confine themselves to two branches of science only.)

#### Geology and Physical Geography.

1. How would you recognise an extinct volcano? What traces of volcanic action are to be observed in the British Isles?
2. What are the chief fossils of the mountain limestone? What beds lie immediately above and what immediately below that rock in the British Isles?
3. Describe the effects of (1) glacial action, (2) earthquakes.

#### Physics.

1. Describe Attwood's machine and explain its use.
2. State the facts which demonstrate that, with the exception of tidal energy, all the work done in the world is due to the sun.
3. Explain the electrical phenomena illustrated and the apparatus necessary in sending an ordinary telegraphic message.

#### Botany.

1. Give the characters of the following natural orders: (1) Primulaceæ, (2) Iridaceæ, (3) Convolvulaceæ, (4) Linaceæ, (5) Polygonaceæ.
Describe the structure of an orchis, and explain the method of fertilisation in that genus.
2. What is the botanical nature of (1) ergot of rye, (2) potato disease, (3) smut of corn), (4) lily disease.
3. Define the following terms: (1) Umbel, (2) spike, (3) capitulum, (4) raceme, (5) placenta, (6) albumen, (7) bract, (8) petiole, (9) sepal, (10) cyme, and give an explanation of each.

#### Zoology.

1. How would you recognise a poisonous snake? Describe the structure of the skull and the anatomy of the poison apparatus in any such snake. What difference of action is there between the poison of the cobra and that of a viper?
2. Name the entozoa which inhabit the human body, and describe fully the structure and development of any one form.
3. Describe the placentation of (1) the elephant, (2) the mare, (3) the cow, (4) the cat; and the dentition of (*a*) the sheep, (*b*) the rabbit, (*c*) the dog, (*d*) the sloth.

A Correction.—A paragraph appeared in The Hospital last week referring to the resignation of several members of the medical staff at the "Guildford Cottage Hospital." This was an error. There is no such institution. The wording should have been "a cottage hospital near Guildford."

